# Midday Depression vs. Midday Peak in Diurnal Light Interception: Contrasting Patterns at Crown and Leaf Scales in a Tropical Evergreen Tree

**DOI:** 10.3389/fpls.2018.00727

**Published:** 2018-05-31

**Authors:** Agustina Ventre-Lespiaucq, Nicola S. Flanagan, Nhora H. Ospina-Calderón, Juan A. Delgado, Adrián Escudero

**Affiliations:** ^1^Area of Biodiversity and Conservation, Department of Biology and Geology, Physics and Inorganic Chemistry, Universidad Rey Juan Carlos, Móstoles, Spain; ^2^Department of Natural Sciences and Mathematics, Pontificia Universidad Javeriana Sede Cali, Cali, Colombia; ^3^Department of Biology, Edificio 320, Ciudadela Universitaria Melendez, Universidad del Valle, Cali, Colombia; ^4^Department of Ecology, Faculty of Biology, Universidad Complutense de Madrid, Madrid, Spain

**Keywords:** crown architecture, crown openness, diurnal course of light interception, foliage clumping, leaf angle, light stress, *Psidium guajava*, temporal variation

## Abstract

Crown architecture usually is heterogeneous as a result of foraging in spatially and temporally heterogeneous light environments. Ecologists are only beginning to identify the importance of temporal heterogeneity for light acquisition in plants, especially at the diurnal scale. Crown architectural heterogeneity often leads to a diurnal variation in light interception. However, maximizing light interception during midday may not be an optimal strategy in environments with excess light. Instead, long-lived plants are expected to show crown architectures and leaf positions that meet the contrasting needs of light interception and avoidance of excess light on a diurnal basis. We expected a midday depression in the diurnal course of light interception both at the whole-crown and leaf scales, as a strategy to avoid the interception of excessive irradiance. We tested this hypothesis in a population of guava trees (*Psidium guajava* L.) growing in an open tropical grassland. We quantified three crown architectural traits: intra-individual heterogeneity in foliage clumping, crown openness, and leaf position angles. We estimated the diurnal course of light interception at the crown scale using hemispheric photographs, and at the leaf scale using the cosine of solar incidence. Crowns showed a midday depression in light interception, while leaves showed a midday peak. These contrasting patterns were related to architectural traits. At the crown scale, the midday depression of light interception was linked to a greater crown openness and foliage clumping in crown tops than in the lateral parts of the crown. At the leaf scale, an average inclination angle of 45° led to the midday peak in light interception, but with a huge among-leaf variation in position angles. The mismatch in diurnal course of light interception at crown and leaf scales can indicate that different processes are being optimized at each scale. These findings suggest that the diurnal course of light interception may be an important dimension of the resource acquisition strategies of long-lived woody plants. Using a temporal approach as the one applied here may improve our understanding of the diversity of crown architectures found across and within environments.

## Introduction

Light heterogeneity in space and time critically affects light interception in plants (Baldocchi and Collineau, 1994). In spatially heterogeneous light environments, plants enhance light interception by directing crown growth toward productive resource patches, such as forest gaps (Ackerly and Bazzaz, 1995; De Kroon et al., 2009). As a result of directional foraging, crown architecture usually is spatially heterogeneous and, consequently, light interception can quantitatively differ between regions of the crown. Because light also varies with time, crown architectural heterogeneity often leads to a temporal heterogeneity in light interception (reviewed in Baldocchi and Collineau, 1994; Cescatti and Niinemets, 2004). Yet, ecologists are only beginning to identify the importance of temporal heterogeneity for resource acquisition in plants, especially below the seasonal scale (Schofield et al., 2018). In plant light interception studies, the temporal dimension at short-term scales is largely acknowledged but frequently summarized in time-integrated measurements (Baldocchi and Collineau, 1994). As an unintended consequence, light interception at shorter time scales, such as the diurnal scale, have received less attention (but see, Valladares and Pugnaire, 1999; Granado-Yela et al., 2011; Escribano-Rocafort et al., 2016; Ventre-Lespiaucq et al., 2017). This is surprising since it is well-known that diurnal variation in light interception has consequences for circadian physiological processes such as photosynthesis and evapotranspiration (Valladares and Niinemets, 2007). In this context, the diurnal dimension of light interception can provide novel insights for understanding the diversity of plant functional responses under the variety of environmental constraints found in nature.

Trade-offs between light interception and water balance provide a key mechanism for the acclimation of plant architecture to light conditions (Niinemets and Valladares, 2004). High irradiance can become limiting for plants growing in open sites, to the point that plants change crown architecture to avoid the interception of excess irradiance (McMillen and McClendon, 1979; Valladares and Pearcy, 1998; Werner et al., 1999, 2001). Light interception can conflict with other plant functions on a diurnal basis (Tuzet et al., 2003; Valladares and Niinemets, 2007), because the diurnal increase in solar radiation often leads to an increase in air temperature and a reduction in air moisture (Sánchez et al., 2014). Therefore, maximizing the exposure to light during midday may not be an optimal strategy in environmental settings with excess light (Valladares and Pearcy, 1999; Valladares and Pugnaire, 1999; Falster and Westoby, 2003). Alternative architectural solutions to this trade-off are expected to affect the diurnal course of light interception (Werner et al., 1999). Crown architecture in long-lived plants, which must endure diurnal environmental fluctuations for decades, may then result from a solution to this trade-off.

Crown architecture results from biological adaptation in evolutionary time scales, as well as from the expression of plastic responses to the particular conditions in which individuals grow (Hallé et al., 1978; Schmid and Bazzaz, 1990; Barthélemy and Caraglio, 2007; Valladares and Niinemets, 2007). Plastic architectural responses involve the adjustment of structural traits at several organizational levels, from the leaf to the whole crown scale (Cescatti and Niinemets, 2004; Barthélemy and Caraglio, 2007). At the leaf scale, the diurnal course of leaf light interception in broadleaved plants is determined mainly by leaf position angles (Herbert, 1983; Pearcy et al., 2005). Horizontal leaf angles maximize the capture of midday light, which is a common phenotype in light-limited understory plants (Valladares and Pearcy, 1998; Pearcy et al., 2005; Ventre-Lespiaucq et al., 2017). By contrast, vertical leaf angles lead to a midday depression of light interception under high light (Falster and Westoby, 2003; Pearcy et al., 2005). In addition, leaf angles can be highly structured within the crown of a single plant, whereby leaves placed in contrasting crown exposures can display different average angles and, consequently, different diurnal courses of light interception (Granado-Yela et al., 2011; Escribano-Rocafort et al., 2016). At the crown scale, traits related to leaf area distribution, such as foliage clumping and leaf density have important consequences for light interception (Wang and Jarvis, 1990; Whitehead et al., 1990; Baldocchi and Collineau, 1994; Duursma et al., 2012). Clumped canopies (i.e., with zones of high and low leaf density) have less light interception efficiency than uniform ones, all other factors being equal, because clumping enhances self-shading (De Castro and Fetcher, 1999; Campbell and Norman, 2000; Delagrange et al., 2006). Although most of the patterns referred above derive from studies of time-integrated light interception, this framework is equally valid for diurnal courses of light interception.

We aimed at assessing how diurnal courses of light interception relate to crown architecture at the leaf and crown scales. As a model, we used the tropical evergreen tree *Psidium guajava* L. In the tropics, solar geometry changes little throughout the year; therefore, evergreens retaining their leaves year-round are expected to show crown structures and leaf positions that warrant an effective light interception for all the year. Since crown architecture is affected by neighboring trees, we selected individuals growing in isolation for studying relationships between structural traits and the temporal dynamics of light interception without the interference of above-ground competition for light. We hypothesized that the heterogeneity in crown architecture optimizes light interception on a diurnal basis. In particular: (1) crown and leaf light interception would show a midday depression, because, although light comes from zenith during almost all the diurnal cycle, high irradiance and heat load at noon may limit physiological processes. (2) At the crown scale, the midday depression of light interception would be attained by heterogeneous crowns with more foliage clumping in the crown tops than in the lateral parts. (3) At the leaf scale, the midday depression of light interception would result from structural photoprotection afforded by vertically-arranged leaves.

## Materials and Methods

### Species and Study Site

The guava (*P. guajava*) is a widespread tropical evergreen tree species native to Mexico, Central and South America and the Caribbean, and widely cultivated and naturalized in these and other tropical and subtropical regions of the world (Stone, 1970; Menzel, 1985). This species grows in both humid and sub-humid climates from the sea level to 2740 m a.s.l. (MPTS database). It is an intermediate shade- and drought-tolerant species (Green, 2009), that grows from full sun to semi-shade conditions with an optimum of annual rainfall between 1000 and 2000 mm (Samson, 1986; Pontikis, 1996). The habit is of a relatively small tree, 3–10 m high, with a highly ramified trunk. Leaves are ovate-elliptic, 3 to 13.5 cm long and 3 to 6 cm wide, with opposite phyllotaxis (Conabio, 2003). Leaves flush several times during the year (Green, 2009) and leaf life span can exceed a year (Martin et al., 1994).

We studied a population of *P. guajava* trees growing in a rural region of the Popayán plateau, Cauca Department, Colombia (2° 23^′^ 28^′′^ N, 76° 39^′^ 24^′′^ W) at 1760 m, adjacent to the Popayán Botanical Garden. The climate is tropical monsoon (Am in the Köppen classification) with a not very pronounced dry season (**Supplementary Table 1**). The landscape consists of tropical sub-Andean forest patches (Cuatrecasas, 1958) and relatively small pastures with sparse crop trees, mainly citrus, but also guava trees. Field work was conducted in November–December 2012 (**Supplementary Table 1**).

### Sampling Design

**FIGURE 1:**
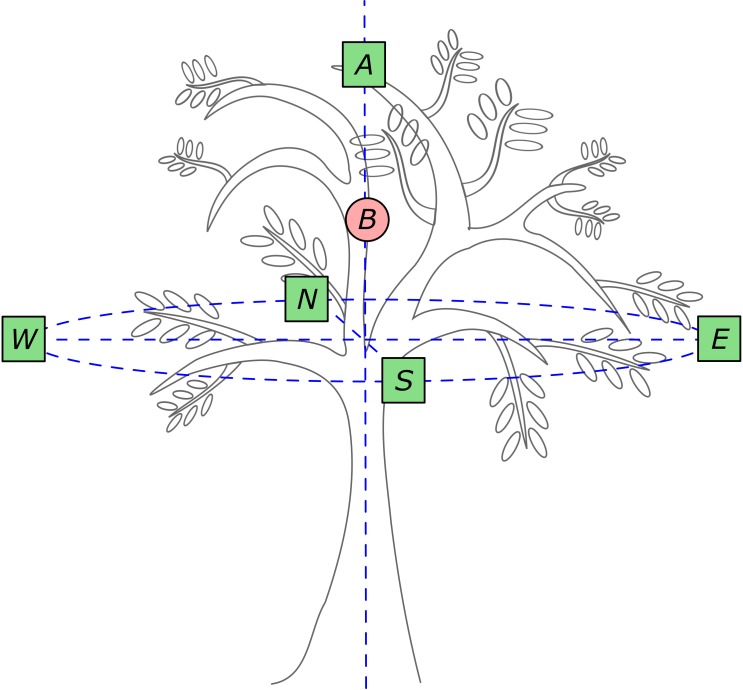
Field sampling design. Crown-scale variables were quantified in crown sector *B*. Leaf-scale variables were quantified in crown sectors *A, N, E, S* and *W*, where 10 leaves per sector were sampled (50 leaves per tree; squares). Hemispheric photographs were taken in the six sampling points. Modified from Hallé et al., 1978.

We randomly selected nine isolated and healthy adult guava trees that averaged 5.45 ± 0.52 m height, and 7.29 ± 0.67 and 6.73 ± 0.68 major and minor crown diameter, respectively. We defined six sampling points in each crown (**Figure 1**). One was located in the center of the crown (point B, **Figure 1**), in the hollow (leafless) central part of the crown at 3.80 ± 0.41 m height above the ground. At this point, we quantified crown-scale variables (see below) with a hemispheric photograph pointing zenith. To investigate leaf-level variables (see below), we defined five sampling points: one immediately above the top leaf layer at maximum tree height (5.45 ± 0.52 m); and the remaining sampling points were located in the four main compass directions (*N, E, S*, and *W*) in the basal exterior region of the crown (**Figure 1**) at 2.77 ± 0.09 m height. In each of these five sampling points we took a hemispheric photograph to assess incident light and sampled the leaf position angles of 10 fully expanded leaves. We sampled 50 leaves per tree and 450 leaves in total.

### Hemispheric Picture Sampling and Analysis

To obtain radiation and crown structure data, we took a hemispheric picture in each of the six sampling points with a Nikon Coolpix 5000 digital camera coupled to a Nikon F8 fisheye lens of 180° field of view (Nikon, Tokyo, Japan) and mounted on a self-leveling system (Regent Instruments, Inc., Canada). All photographs were taken with automatic camera settings and 5 MP resolution, within a single day under overcast sky conditions, so that the distribution of light can be assumed to be uniform or nearly-uniform (Fournier et al., 2017).

Pictures were analyzed with the software WinSCANOPY v.2006b (Regent Instruments, Inc., Canada). Prior to analyses, we customized radiation settings with information on the location studied to improve the estimates of photosynthetically active radiation (PAR, μmol m^-2^ s^-1^). We set atmospheric transmittance to 0.63 and the diffuse to direct PAR ratio to 0.44, as estimated from cloudiness-corrected radiation parameters obtained from satellite climatic data (NASA LaRC Project, 2016). We set diffuse radiation distribution to a standard overcast (SOC) model, which takes into account anisotropy in the distribution of diffuse irradiance in the sky hemisphere (Moon and Spencer, 1942). Pixel classification threshold was set to 131, as obtained from an average (131 ± 1) of two passes of manual thresholding by an experienced user. The sky grid was divided into 24 equiangular azimuth slices and 10 zenith elevation rings (240 crown segments in total, Gonsamo et al., 2010). The lowest zenith ring (90–81°) was discarded for analyses since it is often affected by optical errors (Gonsamo et al., 2013), thus we finally used 216 crown segments.

### Crown-Scale Architecture

We sought to find a structural correlate of diurnal crown light interception. We used two indicators of crown structure as obtained from hemispheric picture analysis: the gap fraction and the foliage clumping index. The gap fraction is the area of the picture not obstructed by vegetation. It provides information on crown structure (Gonsamo et al., 2013). The clumping index is a measure of foliage distribution within the crown space. We estimated the clumping index based on gap size distribution (Leblanc et al., 2005), since it performs better in structure analysis than other approaches (Gonsamo and Pellikka, 2009). Light transmittance estimated with hemispheric pictures is obviously correlated with the gap fraction and clumping index of the pictures. To avoid spurious correlations, instead of using a global estimate per picture, we obtained gap fraction and the clumping index for each of the 216 crown segments and focused on their within-crown variation. In particular, we inspected their variance across crown segments (see section “Data Analyses”) and calculated the coefficient of variation of the clumping index across the 216 crown segments.

### Leaf-Scale Architecture

We used the silhouette to leaf area ratio (STAR; %) as a proxy of leaf light interception. To obtain STAR, we took *in situ* measurements of three leaf angles describing leaf position relative to the Earth’s magnetic and gravitational fields, namely pitch (angular elevation relative to the horizontal), roll (left–right rotation angle around leaf’s midrib) and lamina course (azimuth orientation of the vector normal to the leaf lamina surface), using a smartphone (Nokia N86, Nokia Group, Espoo, Finland) with the app Ahmes 1.0 (Escribano-Rocafort et al., 2014). All these recordings were performed by placing the device parallel to the leaf surface during days without wind. We calculated STAR using the package ‘leafSTAR’ in R v3.4.1 (Ventre-Lespiaucq and Santamaría, 2017), which implements the equations in Escribano-Rocafort et al. (2014). We estimated the leaf tilt angle relative to the horizontal plane (0° horizontal, 90° vertical) using leaf pitch and roll angles (Eq. 1 in Escribano-Rocafort et al., 2014). Leaf tilt and course angles combined with the geographic coordinates of the population, and information on local hour and date were used to calculate the silhouette area of the leaf blade (SAL, %) with Eq. 3 in Escribano-Rocafort et al. (2014). This equation uses the cosine law to derive the proportion of a one-sided surface that is exposed to directional irradiance at a given location and time. SAL obtained in this way is equivalent to the leaf’s silhouette to total leaf area ratio (STAR) multiplied by 100, and we will refer to it as STAR hereafter. Although STAR does not contemplate leaf overlapping nor leaf clumping, it allows an upper theoretical limit of leaf potential exposure to directional light to be established (Granado-Yela et al., 2011).

### Diurnal Courses of Light Interception

We assessed crown diurnal light interception as the percentage of incident PAR intercepted (PARi) by the crown at crown sector *B* per hour, estimated as (PARs–PARt) × 100, where PARs is incident PAR and PARt is PAR transmitted to crown sector *B*, every hour from 0600 to 1800 local time with noon at 1200 (UTC -5), every 4 days during the year 2012. We used contour plots to investigate the pattern of diurnal variation in light interception for the average day of each month of 2012 (**Supplementary Figure 1**). The inspection of monthly data was intended to confirm whether the diurnal patterns were constant throughout the year. Since the analyses are based on one-time pictures, any pattern of monthly variation is due to the current spatial configuration of the crown and changes in solar apparent position, and not to seasonal changes in trees’ vegetative structure.

Leaf STAR varies diurnally in bi-facial, static leaves (i.e., that do not track the solar apparent movement) as a result of solar geometry effects, with a diurnal peak and valley of surface exposure to directional light. To relate diurnal courses of STAR to the diurnal course of light, we calculated diurnal STAR using Eq. 3 in Escribano-Rocafort et al. (2014) along with the diurnal course of total PAR per crown sector every hour from 0600 to 1800 (UTC -5), for the average day of December of 2012.

### Data Analyses

Data analyses were structured into two parts. We first described the diurnal patterns of crown- and leaf-scale light interception, and then we dissected the structural correlates. All analyses were performed in R v3.4.1 (R Core Team, 2017).

We investigated whether leaf STAR and crown light interception were coupled to the diurnal course of incident PAR using a linear mixed models (LMMs) approach. We generated a set of fixed-effects models including the tree identity as a random determinant (Bolker et al., 2009) and performed a model selection based on Akaike’s Information Criterion (AIC) with the ‘nlme’ package (Pinheiro et al., 2017). We assessed the structure of the random effects through restricted maximum likelihood estimations, and then choose the structure of the fixed effects through maximum likelihood estimations. Two models were considered to differ when AIC difference (ΔAIC) was above 10 (Burnham and Anderson, 2002). We first assessed visually whether the pattern of diurnal STAR varied between crown sectors. Since the patterns were similar (see **Supplementary Figure 2A**), we averaged diurnal STAR over all leaves per tree and modeled it as a function of three continuous predictors: incident PAR, the hour of the day and tree height as a co-variable, with tree identity included as a random factor. In an additional model, we assessed crown light interception as a function of incident PAR and hour of the day, tree height as a co-variable and tree identity in the random term.

We assessed whether the clumping index varied vertically across zenith elevation rings and horizontally across azimuth slices by means of linear regressions. Since the clumping index did not vary substantially between azimuths (not shown), we pooled azimuth slices per zenith ring. To describe the distribution of foliage within the crown in general terms, we correlated gap fraction with the clumping index across zenith rings. Zenithal changes in the variance of both indices were visually inspected with a bivariate plot. In addition, we wanted to have an approximate idea of how spatial heterogeneity in foliage clumping affected whole-plant light interception in the long term. For this, we calculated whole-plant annual light interception as the annual cumulative PARi/annual cumulative PARs × 100 and performed a linear regression using the coefficient of variation of the clumping index (CI_CV_) as a predictor, and tree height as a co-variable. The CI_CV_ was calculated over the 216 crown segments.

To determine whether leaves converged in STAR peaks at certain hours of the day, or alternatively, whether STAR peaked erratically at different hours, we investigated the statistical distribution of leaf angles using circular statistics in the package ‘circular’ (Agostinelli and Lund, 2017). In particular, we estimated two parameters describing the frequency distribution of leaf tilt and course angles within each crown sector. The central statistic is the average angular direction (μ), which is a circular analog to the arithmetic mean (Ruxton, 2017). The other descriptor is the circular dispersion parameter (κ), an estimator of the concentration of the data in the whole range of values. The higher this value, the greater the dispersion of the data (Ruxton, 2017). To assess whether leaves oriented their lamina toward a preferred azimuth direction, we tested whether the statistical distribution of leaf course angles in each sector fitted a von Mises distribution (the circular analog to the parametric Gaussian distribution) with a mean angular direction at four alternative orientations: N (0 and 360°), E (90°), S (180°) and W (270°), and estimated 95% confidence intervals with bootstrapping methods following Ruxton (2017). To assess whether leaves oriented their lamina toward a preferred zenith direction, we tested whether the statistical distribution of leaf tilt angles in each sector fitted a von Mises distribution without making *a priori* assumptions about the value of the mean angular direction. We tested whether the parameters μ and κ differed between sectors with Rao’s test (Pewsey et al., 2013).

## Results

**FIGURE 2:**
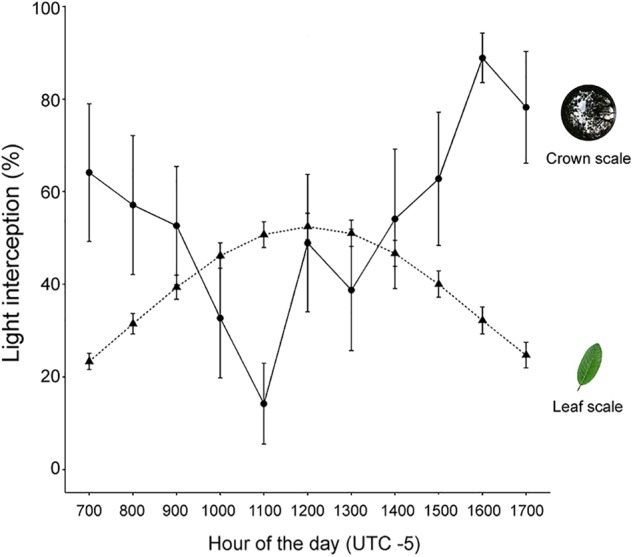
Diurnal courses of light interception at the crown and leaf scales. Crown scale light interception is the percentage of incident PAR intercepted by the crown. Leaf scale potential light interception is represented with STAR (%). Bars denote the SE of the mean for *N* = 9 trees.

Crown light interception and leaf STAR exhibited opposite diurnal patterns (**Figure 2**). Crown light interception showed a midday depression while leaf STAR showed a midday peak (**Figure 2**). The negative relationship between crown light interception and incident PAR was weak (*R*^2^ = 0.14) but significant at α = 0.05 (*p* < 0.001). Crown light interception decreased by a 39.7% from hours of low PAR (7:00, 17:00; **Figure 2**) to the hour of peak PAR (12:00) (slope = -0.019 ± 0.005). The remaining predictors (leaf STAR), hour of the day, and tree height did not contribute substantially to explain variance in crowns’ light interception (*p* > 0.1) neither did the random term (i.e., tree identity, ΔAIC = 2.2).

Leaf STAR increased by a 27.3% with increasing incident PAR (slope = 0.014 ± 0.001). The *Tree* in the random term contributed to explain variance in STAR (*Tree* = 2.4, residual variance = 2.3). Because *R*^2^ and *p*-values are not reliable within the mixed-effects modeling framework (Bolker et al., 2009), we cannot ascertain whether the relationship between STAR and PAR is statistically significant. Nevertheless, when we compared models with different fixed-effect structures, we found evidence supporting an effect of diurnal PAR on diurnal STAR. Models containing PAR among predictors had by far more explanatory power than models without it (e.g., ΔAIC = 122.4). Likewise, the best model according to the AIC criterion (STAR as a function of PAR) had more explanatory power than the saturated model (ΔAIC = 11.8) or a null model (ΔAIC = 133.6).

**FIGURE 3:**
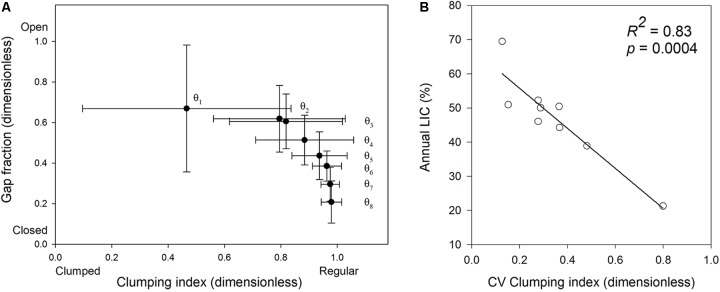
Relationship between the clumping index and the gap fraction across zenith rings **(A)**. Closed circles represent each of 8 zenith regions spanning 9° elevation angles, indicated with 𝜃. 𝜃_1_ is the range between 0 and 9° elevation angles (zenith, top central part of the crowns in the picture) and 𝜃_8_ is the range between 72 and 81° (horizon, lateral parts of the crown in the picture). Bars denote 1 standard deviation for *N* = 9 trees. **(B)** Correlation between the CV of the clumping index and annual light interception. CV, coefficient of variation calculated over 216 crown segments. *N* = 9 trees.

Crowns showed a heterogeneous spatial structure (**Figure 3A**). The gap fraction and the clumping index almost did not vary across azimuth slices (not shown) but varied across zenith rings (**Figure 3A**), indicating “doughnut-shaped” crowns. The most zenithal part of the photographs was on average 46.1% more open and 51.4% more clumped than the most horizontal (lateral) part (**Figure 3A**). There was some among-tree variation in this pattern. The SD of the gap fraction and clumping index (**Figure 3A**) decreased in a 20% toward the horizontal part of the picture (*R*^2^ = 0.94; *p* = 0.04 and *R*^2^ = 0.99; *p* = 0.001, respectively), showing that the trees were similar in the structure of the lateral parts of the crowns, and differed in the structure of the top parts.

We summarized within-crown structural variation using the CV of the clumping index (CI_CV_), and regressed it against annual crown light interception (**Figure 3B**). In trees with a low CI_CV_ (≈0.18), annual light interception was on average a 40% higher than in the tree with the highest CI_CV_ (0.80; **Figure 3B**). Since the tree sample with the highest CV value may be an influential point (no values between 0.48 and 0.8), we repeated the analysis with *N* = 8 by excluding this tree to see whether this point was influential in the model fit. As a result, the strength of the correlation fell from *R*^2^ = 0.83 to 0.65, but it remained significant at *p* = 0.016. Tree height did not affect annual light interception (*R^2^* = 0.08, *p* = 0.4) or CI_CV_ (*R^2^* = 0.34, *p* = 0.1).

**FIGURE 4:**
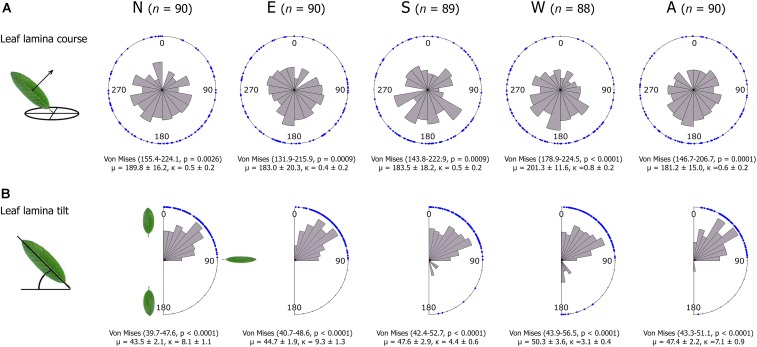
Rose diagrams for the distribution of leaf lamina course **(A)** and tilt angles **(B)** per crown sector. Course angles are shown in degrees clockwise from North (0°), and tilt angles range from a vertical leaf position with the leaf tip pointing toward zenith (0°), to horizontal (90°), and to vertical with the leaf tip pointing downward (180°). The bins represent the frequencies in each direction and blue dots show single leaf data. For leaf course, 95% confidence intervals of the mean direction of the population and their *p*-values correspond to an underlying Von Mises distribution with the mean course direction predicted to be at 180°. For leaf tilt, the underlying distribution was a Von Mises without a specific prediction for the mean direction. For each crown sector, we indicate the number of leaves sampled (*n*), and mean direction (μ), and concentration parameter (κ) with their standard errors. N, E, S, W are the four basal crown sectors in the main compass directions, and A is the central upper crown sector.

Variance in STAR was linked to a large variation in the frequency distribution of leaf course and tilt angles (**Figure 4**). The average leaf course was south (μ in **Figure 4A**), but the values of the concentration parameters κ evidenced a high dispersion of the data (**Figure 4**). High κ values suggest that leaf course may have a multimodal distribution (i.e., leaves’ surfaces can show more than two average orientations). We tested whether leaf course was multimodal by fitting three additional models with expected mean directions at north, east and west (0, 90, and 270°, respectively), but all were non-significant at *p* > 0.4, supporting the south as the main leaf orientation. All crown sectors showed similar μ and κ values (**Figure 4A**) (Rao’s statistic_μ_ = 1.60, *p* = 0.8; and Rao’s statistic_κ_ = 2.58, *p* = 0.6, respectively).

The average leaf tilt angle was 45° relative to the horizon (μ in **Figure 4B**). Leaf tilt angles were concentrated between 0 and 90°, and only few of them fell between 90 and 180° (**Figure 4B**). The low occurrence of leaf tilt angles between 90 and 180° (leaf tips pointing downward) likely indicates an absence of leaf wilting (turgor loss), evidencing the trees were not water-stressed during the sampling period (Salter and Goode, 1967, p. 8). The average tilt angle did not differ between crown sectors (Rao’s statistic_μ_ = 4.74, *p* = 0.3), but the concentration parameters did (Rao’s statistic_κ_ = 17.50, *p* = 0.002), being lower in the west than in the remaining sectors (**Supplementary Table 2**).

## Discussion

We expected to find a midday depression in light interception both at the crown and leaf scales, as a strategy to avoid the interception of excessive irradiance. Our results partially matched these expectations. While crowns indeed showed a midday depression in light interception, leaves showed a patent midday peak in light interception. These contrasting patterns were related to architectural traits, as predicted. At the crown scale, the midday depression of light interception was linked to a greater crown openness and foliage clumping in crown tops and a more regular foliage distribution in the lateral parts of the crown. Although crown tops showed some among-individual variation in the degree of foliage clumping, all individuals showed the pattern of midday depression in light interception. At the leaf scale, an average inclination angle of 45° led to the midday peak found in potential light interception, which departs from our predictions. Nevertheless, among-leaf variation in position angles was huge, pointing to a large variation in the diurnal course of potential light interception at the leaf scale. The mismatch in diurnal course of light interception at crown and leaf scales can indicate that different processes are being optimized at each scale.

All the adult guava trees studied in a tropical mountain site displayed a consistent midday depression in light interception at the population level, as predicted by our first hypothesis. To the best of our knowledge, a midday depression in light interception at the whole-crown scale has only been found in one study (Charbonnier et al., 2013). These authors found that coffee plants growing in open sites in the humid tropics showed a midday depression in light interception. When coffee was surrounded by partially shading trees, the diurnal course of light interception shifted to show a midday peak. The structural correlates of such shift were not evaluated (Charbonnier et al., 2013), but their findings suggest that light and co-varying factors probably reached stressful midday levels in such open sites. This midday depression in light interception may be a frequent response under stressful conditions due to excessive light. However, other studies have found a constant diurnal light interception in other ecosystems and tree species. For instance, peach cultivars growing in temperate locations (Génard and Baret, 1994) and wild *Olea europaea* trees growing in Mediterranean locations (Ventre-Lespiaucq et al., 2016) showed a constant diurnal course of light interception. This suggests that the diurnal course of light interception at the crown level can be species-specific to some extent, because species may show different tolerances to midday excess light (Werner et al., 1999; Valladares and Niinemets, 2007).

The midday depression in light interception at the crown level was linked to particular architectural traits. Hemispheric photograph analyses suggested a doughnut-like crown, where higher zenith rings (near zenith) showed a more open and clumped foliage than lower zenith rings (near the horizon). Conifers in Finland showed a regularly-distributed foliage at the crown base and clumped foliage at the top (Stenberg, 1998), similar to *P. guajava*. However, the meaning of this doughnut-like crown architecture may differ across latitudes. At high latitudes, where the solar elevation angle is low, foliage allocation to crown tops will likely be selected against since top leaves would seldom receive direct sunlight (Kuuluvainen, 1992; Stenberg, 1998; Cescatti and Niinemets, 2004). At low latitudes, allocating foliage to crown tops may also be selected against for a different reason: the year-round vertical incidence of sunlight can result in excess radiation and heat loads on top-crown foliage, entailing a risk for photosynthesis. While it is known that contrasting crown architectures of coexisting plants can be equally functional in terms of light interception efficiency (Valladares et al., 2002), evidence above suggests that similar architectural traits can be functional under different environments. Looking at crown architectures from the perspective of diurnal light interception, as done here, may yield a better understanding of the crown architectural diversity found across and within environments.

Within the common pattern of midday depression in light interception, crown architectural traits showed some variation among guava trees. The structure of the lateral parts of the crowns was similar, while the top parts differed among trees; namely, the tops were more clumped and open in some individuals than in others. These among-tree differences were not enough to blur the common pattern of midday depression of light interception, but they did have consequences for annual integrated light interception. Estimates of annual light interception revealed that trees with more closed-top crowns would intercept more light on an annual basis than trees with more open-top crowns. These annual differences can be explained by the fact that trees filling the crown surface more regularly can increase the capture of light coming from all directions (Delagrange et al., 2006). This suggests that heterogeneity in crown architecture determines annual light interception via differences in the diurnal course of light interception.

Our first hypothesis also anticipated that leaves would show a midday depression in light interception; however, they did not match this expectation. Instead, a midday peak in potential light interception was found. This is an unexpected result for a plant growing in open sites, because light capture beyond photosynthesis saturation comes at a cost for the leaf in the form of water losses and photoinhibition (Pearcy et al., 2005). While maximum light interception (i.e., a 100% STAR during midday) in our study site would require a leaf angle of approximately 23° above the horizon during the solstice of December, the observed average leaf inclination was 45°, which reduced midday leaf exposure by a 50%. In addition, midday incident light peaked at 1000–2000 μmol m^-2^ s^-1^ (**Supplementary Figure 2B**), which can far exceed the light saturation point of *P. guajava* (Singh and Singh, 2007). The leaves of *P. guajava* in this population on average showed a peak light interception during midday, but avoided photoinhibition risk by exposing only a 50% fraction of their surface area directly to light.

The lack of differences in leaf angles or STAR among crown sectors contrasts with findings in other tree species, where leaves adjust their angles to within-crown gradients in irradiance (Werner et al., 2001; Uemura et al., 2006; Granado-Yela et al., 2011; Rubio de Casas et al., 2011). In wild *Olea europaea* trees all leaves within a sector had concurrent position angles that varied continuously along the four crown azimuthal sectors (N, E, S, and W). Such structured variation in leaf angles was interpreted as a way to optimize whole-crown light interception under the particular environmental challenges imposed by the local climate (Escribano-Rocafort et al., 2016, 2017). In *P. guajava*, individual leaves showed a wide variety of orientations and therefore, STAR peaked somewhat erratically. A lack of concert in leaf angles suggests that leaves do not respond to the local light irradiance at the population level but to each particular leaf light micro-environment (Posada et al., 2009; Osada and Hiura, 2017). This result is in line with the high variability in light conditions in this location (**Supplementary Table 1**), where the prevalence of multidirectional light (diffuse light) would reduce the benefit of adopting a restricted range of leaf orientations (Roderick et al., 2001). Our results pinpoint that the structured variation of leaf position angles described in other species is not necessarily widespread. The implications of within-individual variation (*sensu* Herrera, 2017) remain as a poorly studied topic in plant resource acquisition. In particular, understanding the structure of within-individual variation in leaf angles can provide valuable information on how plants cope with environmental light heterogeneity.

We found a mismatch in the diurnal course of light interception between crown and leaf scales, contrary to our prediction. The diurnal course of light interception could differ between scales if it resulted from the optimization of different processes. It has been proposed that plants should maximize light interception at the whole-crown scale but photosynthetic light use efficiency at the leaf scale (Posada et al., 2012). For example, in high light environments, foliage discontinuities of open-top crowns reduce the vertical gradient in irradiance within the crown. This leads both to a reduction in the radiation load upon the most exposed leaves and to a more homogeneous distribution of light among leaves in multi-layered crowns (Horn, 1971). In turn, leaves can maximize light use efficiency, but not necessarily light interception, by finely tuning leaf angle and maximum photosynthesis under the particular light conditions experienced by each leaf (Posada et al., 2009, 2012). From a functional approach, studying crown-scale architectural traits seems capital to understand how plants maximize light interception. Furthermore, a dynamic view that includes the diurnal course of light interception can improve our understanding on how plants integrate different functions across levels of organization.

Our study suggests that the diurnal course of light interception may be an important dimension of the resource acquisition strategies of long-lived woody plants. This raises the opportunity to explore crown structural–functional relationships from a novel perspective, which explicitly includes the diurnal course of incident light and light interception. Currently, many tools are available for assessing the diurnal course of light interception. We chose hemispheric photography because it allows a cost- and time-effective assessment of 2-D crown architecture in large trees. More sophisticated methods already used in smaller plants, such as the analysis of 3D-digitized plants (Pearcy and Yang, 1996), as well as terrestrial laser-scanning (Lim et al., 2003) and 3D growth simulations using structural–functional models (Sterck and Schieving, 2007) will be useful to this end in the near future. Their application using a temporal approach as the one used here may improve our understanding of the diversity of crown architectures found across and within environments.

## Author Contributions

AV-L conceived and designed the study, analyzed the data, and wrote the first draft of the manuscript. AV-L, NF, and NO-C performed field work. All authors discussed results and wrote the manuscript.

## Conflict of Interest Statement

The authors declare that the research was conducted in the absence of any commercial or financial relationships that could be construed as a potential conflict of interest.
