# Supplementary material for: Midday Depression vs. Midday Peak in Diurnal Light Interception: Contrasting Patterns at Crown and Leaf Scales in a Tropical Evergreen Tree
Source: Front Plant Sci. 2018 May 31;9:727. doi: 10.3389/fpls.2018.00727 (PMC5990892; doi:10.3389/fpls.2018.00727)
Supplement: Supplementary file 1 [file Image_1.PDF]

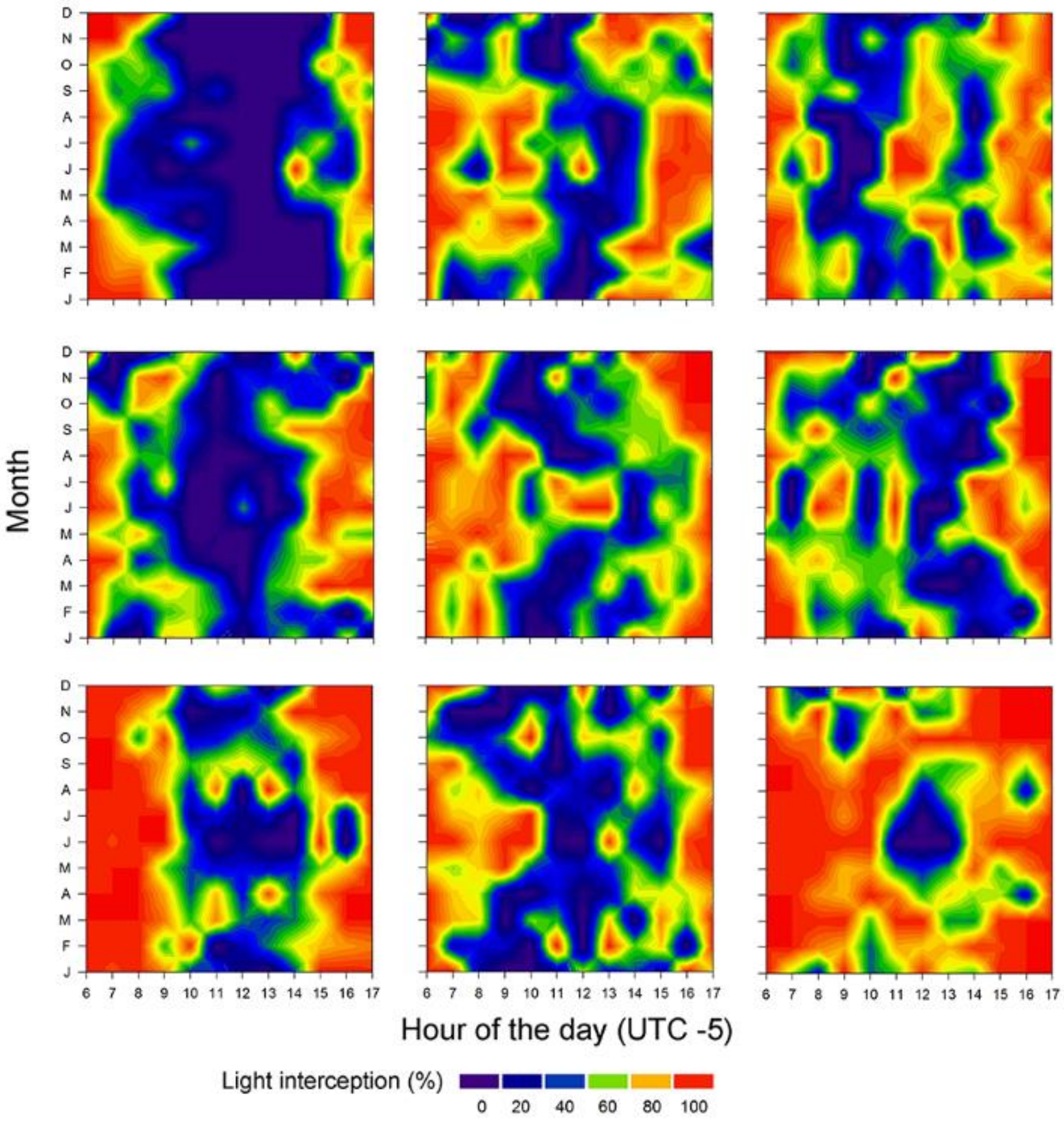

Supplementary Figure 1. Contour plots showing diurnal crown light interception (%) throughout the year 2012 in each of 9 guava trees.
