# Supplementary material for: Midday Depression vs. Midday Peak in Diurnal Light Interception: Contrasting Patterns at Crown and Leaf Scales in a Tropical Evergreen Tree
Source: Front Plant Sci. 2018 May 31;9:727. doi: 10.3389/fpls.2018.00727 (PMC5990892; doi:10.3389/fpls.2018.00727)
Supplement: Supplementary file 2 [file Image_2.PDF]

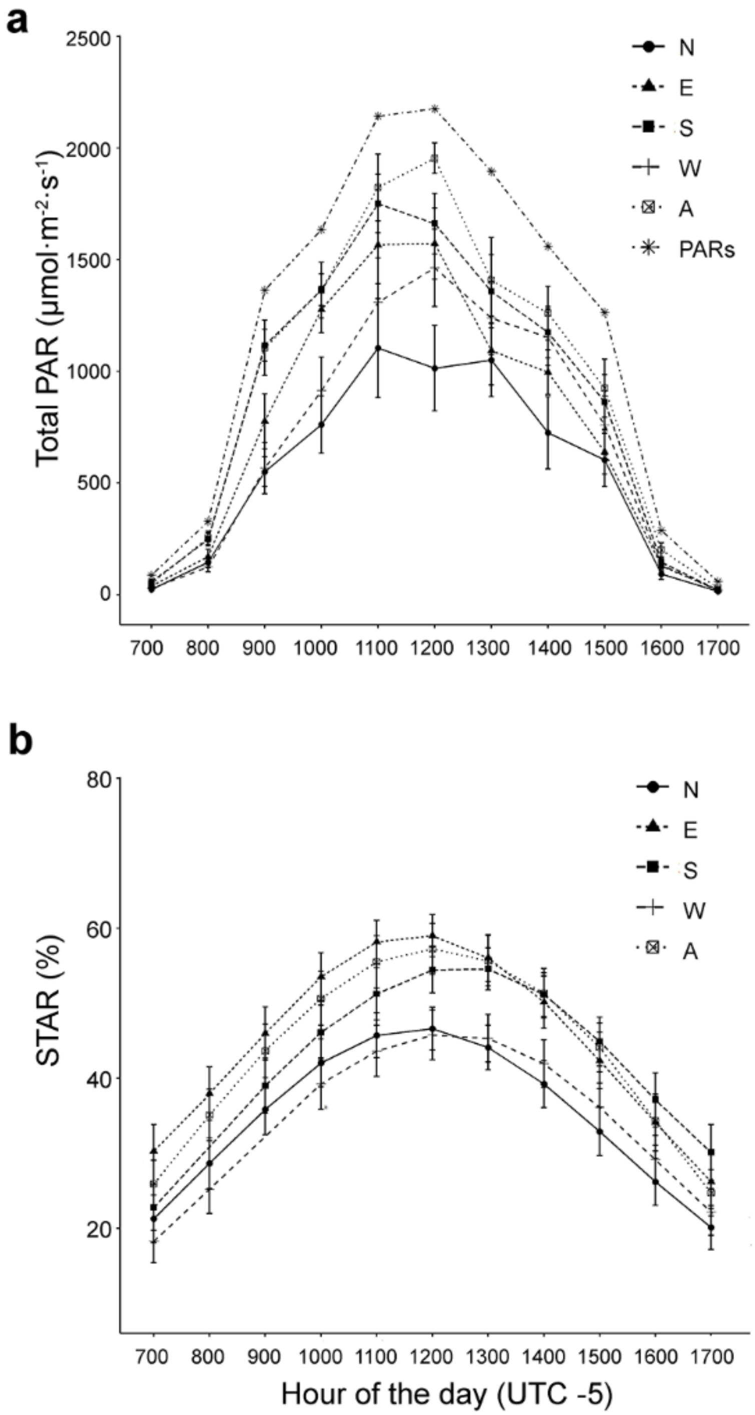

Supplementary Figure 2. Average diurnal courses of clear sky total PAR (**a**) and leaf STAR (**b**) per crown sector (N, E, S, W, and A). PARs denotes theoretical sky PAR incident on a horizontal surface at the study site. Bars denote standard errors for $N = 9$ trees.
