# Supplementary material for: Midday Depression vs. Midday Peak in Diurnal Light Interception: Contrasting Patterns at Crown and Leaf Scales in a Tropical Evergreen Tree
Source: Front Plant Sci. 2018 May 31;9:727. doi: 10.3389/fpls.2018.00727 (PMC5990892; doi:10.3389/fpls.2018.00727)
Supplement: Supplementary file 3 [file Table_1.PDF]

Supplementary Table 1. Monthly and annual averages for climatic variables at the study site, for the period 1983-2005 (obtained from the NASA LaRC POWER Project; https://eosweb.larc.nasa.gov/sse/). T: Monthly average temperature; Tm: Monthly average of the minimum temperatures; TM: Monthly average of the maximum temperatures; RH: Relative Air Humidity; R: Rainfall. CC: Cloud Cover; IA: Percentage of monthly average Insolation Attenuated by clouds.

**Month**	**T (ºC)**	**Tm**	**TM**	**RH (%)**	**R (mm day^{-1})**	**Monthly average CC (%)**			**IA (%)***
						< 10 %	***10 - 70 %***	***>70 %***	
Jan	18.5	16.1	21.2	81.2	4.18	0.88	25.03	74	34.03
Feb	18.8	16.3	21.6	80.4	4.75	1.39	19.97	78.57	35.33
Mar	19	16.5	21.8	80.8	5.39	2.91	23.55	73.45	35.78
Apr	19	16.6	21.5	81	7.21	1.48	21.21	77.23	36.84
May	18.8	16.3	21.3	78.3	6.29	1.61	21.28	77.05	35.45
Jun	18.3	15.8	20.9	74.8	4.84	3.45	27.18	69.3	31.49
Jul	18.7	15.7	21.8	64.9	3.59	3.98	29.78	66.18	29.07
Aug	19.7	16.3	23.1	58.4	3.1	4.31	27.48	68.1	32.28
Sep	20	16.8	23.2	62.1	5.01	1.48	21.62	76.8	36.25
Oct	19.4	16.8	22.3	71.7	6.48	0.84	19.98	79.08	37.09
Nov	18.6	16.3	21	81	6.42	1.37	21.58	76.95	36.2
Dec	18.5	16.3	20.9	82.8	4.76	2.71	27.4	69.83	34.16
Annual	**18.9**	**16.3**	**21.7**	**74.7**	**5.16**	**2.2**	**23.84**	**73.88**	**34.48**

* IA = 100 × (PI – PIc) × PI^{-1}. PI: monthly average Potential Insolation incident on a horizontal surface (kWh·m^{-2}·day^{-1}); PIc: PI corrected for the clearness index, which accounts for cloudiness.
