# Supplementary material for: Midday Depression vs. Midday Peak in Diurnal Light Interception: Contrasting Patterns at Crown and Leaf Scales in a Tropical Evergreen Tree
Source: Front Plant Sci. 2018 May 31;9:727. doi: 10.3389/fpls.2018.00727 (PMC5990892; doi:10.3389/fpls.2018.00727)
Supplement: Supplementary file 4 [file Table_2.PDF]

Supplementary Table 2. Rao's test summary for differences between crown sectors in the concentration parameter κ. N: North; E: East; S: South; W: West; A: crown-top.

**Crown sectors**	**Rao's statistic**	***p***
N, E, A	0.90	0.639
S, W	1.61	0.205
N, E, A, S	7.16	0.067
N, E, A, W	11.68	0.009
